# 2188: Implanted multijoint functional electrical stimulation assistance improves walking efficiency after stroke: A case report

**DOI:** 10.1017/cts.2017.208

**Published:** 2018-05-10

**Authors:** Nathaniel Makowski, Rudi Kobetic, Lisa Lombardo, Kevin Foglyano, Gilles Pinault, Stephen Selkirk, Ronald Triolo

**Affiliations:** Case Western Reserve University, Cleveland, OH, USA

## Abstract

OBJECTIVES/SPECIFIC AIMS: Evaluate the effect of multijoint functional electrical stimulation (FES) on energy consumption during post-stroke walking. METHODS/STUDY POPULATION: A 67-year-old male with chronic stroke was implanted with an 8-channel implanted pulse generator to stimulate flexor and extensor muscles of the hip, knee, and ankle. Oxygen consumption was measured with a k2b4 portable pulmonary gas analyzer during walking with and without FES assistance. Data were analyzed during steady state oxygen consumption within the last 2 minutes of a 5 minute walk. Distance and walking speed were also measured. RESULTS/ANTICIPATED RESULTS: Electrical stimulation increased walking speed from 0.29 to 0.64 minute/second. Faster walking corresponded with increased oxygen consumption from 10.1 to 14.4 mL O_2_/kg per minute. Energy cost, consumption as a function of distance, decreased from 3.7 to 2.9 mL O_2_/kg per minute walking with stimulation compared with without. DISCUSSION/SIGNIFICANCE OF IMPACT: These preliminary data suggest improvements in walking speed with FES are accompanied by increased energy consumption and decreased energy cost. Oxygen consumption during FES assisted walking was <50% of the peak for able bodied individuals of similar age; patients may successfully use the system for community ambulation.

